# Organized Lung Cancer Screening of Subjects Occupationally Exposed to Lung Carcinogens: The LUng Cancer Screening Occupational exposure Study Entry Findings

**DOI:** 10.1016/j.jtocrr.2026.100995

**Published:** 2026-03-28

**Authors:** Fleur Delva, Sébastien Gendarme, Luca Boudet, Maeva Zysman, Claire Fuhrman, Pierre-Yves Brillet, Patrick Brochard, Christophe Paris, Olivier Bylicki, Véronique Le Denmat, Brice Loddé, Milia Belacel, Nadia Abdessemed, Murielle Despagne, Kellian Jaunas, Aude Lacourt, Bénédicte Clin, Jean-François Gehanno, François Laurent, Gael Dournes, Simone Mathoulin-Pélissier, Christos Chouaïd, Jean-Claude Pairon

**Affiliations:** a Epicene, INSERM U1219, University Bordeaux, France; b Service Santé Travail Environnement, CHU de Bordeaux, Bordeaux, France; c Inserm CIC1401, Clinical and Epidemiological Research Unit, Bordeaux, France; d Service de Pneumologie et Oncologie Thoracique, APHP Hôpital Tenon et Université Paris-Est-Créteil, Inserm, IMRB (CEpiA Team), Créteil, France; e Institut Santé-Travail Paris-Est, Centre Hospitalier Intercommunal de Créteil, France; f Service de pathologies professionnelles et de l’environnement, Centre Hospitalier Intercommunal de Créteil, France; g Institut Lyric, Université de Bordeaux-Inserm U1045, Pessac, France; h Service de Pneumologie, CHI de Créteil, UPEC, Créteil, France; i Service de radiologie, Hôpital Avicenne, INSERM 1272, Université Paris 13, Bobigny, France; j Service de Maladies Professionnelles, CHU de Rennes, Rennes, France; k ESTER, INSERM U1085, Rennes, France; l Service de Pneumologie, HIA Sainte-Anne, Toulon, Ecole du Val de Grace, Paris, France; m Centre de Pathologies Professionnelles et Environnementales, CHU de Brest, Brest, France; n Laboratoire d’Etudes et de Recherche en Sociologie, LABERS EA 3149, Université Brest, Brest, France; o INSERM U1086 “ANTICIPE,”, Caen, France; p CHU de Caen, Service de santé au travail et pathologie professionnelle, Caen, France; q Institut de maladies professionnelles, CHU de Rouen, France; r Department of Cardiovascular Imaging, CHU de Bordeaux, Pessac, France; s CIC1401, Epidémiologie et recherche clinique, Institut Bergonié, Bordeaux, France

**Keywords:** Lung cancer, Screening, Occupational exposure, Prevention

## Abstract

**Introduction:**

The LUng Cancer Screening Occupational exposure (LUCSO) program was designed as a feasibility study of organized screening for lung cancer in subjects with occupational exposure to lung carcinogens in France. Herein, we report the program’s entry-level (baseline) findings.

**Methods:**

The inclusion criteria were subjects aged 55 to 74 years, with sufficient exposure to International Agency for Research on Cancer group 1 occupational carcinogens and smoker or ex-smoker for less than 15 years. The first phase of the study addressed subjects aged 65 to 74 years in two French regional counties to test feasibility. Lung cancer screening was based on a low-dose computed tomography (CT) scan at baseline and spirometry, followed by an annual CT scan for 2 years and telephone follow-up at 3 years.

**Results:**

Letters were sent to 247,152 subjects from the Regional Cancer Screening Coordination Center inviting them to participate, allowing eligibility screening by letter. Among the 21,502 respondents (8.7%), 311 (1.4%) were included. Asbestos exposure predominated (92%), but co-exposures were also frequent (73% crystalline silica and 48% welding fumes); 45% were active smokers at inclusion, and 37% of them agreed to consult a smoking-cessation specialist. Baseline CT scans revealed anomalies in 19 (6.1%); seven (2.4%) were diagnosed with the following stages of lung cancer: three stage IA, three stage IIA, and one stage IIB; the false-positive rate was 4.2%.

**Conclusion:**

Before considering nationwide implementation, this feasibility study provided new real-life insights finding that the selected population was indeed at high risk of lung cancer and that screening effectively detected early stages.

## Introduction

In 2022, with 2.5 million new cases worldwide, lung cancer was the most common cancer. It was also the leading cause of cancer-related deaths, accounting for almost one in five deaths.^1^ Apart from smoking, occupational and environmental exposure(s) to carcinogens are major risk factors for lung cancer. The International Agency for Research on Cancer (IARC) classified 21 carcinogens or exposures of occupational origin (i.e., definite carcinogens) in group 1 and associated them with an excess of lung cancers. In France, it was recently estimated that 14.6% of lung cancers are attributable to occupational exposure (19.3% in men and 2.6% in women).^2^ Asbestos is the most frequently implicated agent.^3^

Results of the randomized clinical American National Lung Screening Trial (NLST) revealed the efficacy of lung cancer screening with an annual low-dose chest computed tomography (CT) scan for smokers with more than 30 pack-years (PY) or former smokers who had quit smoking less than 15 years earlier.^4^ Those results were further confirmed by the 2020 publication of the NEderlands Leuvens Screening ONderzoek trial (NELSON; Belgium, The Netherlands).^5^ Since the NLST publication, several recommendations and expert opinions have been published.^6^, ^7^, ^8^, ^9^, ^10^, ^11^, ^12^, ^13^, ^14^, ^15^ Most recommended the introduction of lung cancer screening with low-dose thoracic CT scans, albeit under strict, supervised conditions. Until relatively recently, the relevance of such screening, evaluated in populations of smokers, had not been the subject of specific studies in populations exposed to occupational carcinogens. However, as of 2012, it was reported that occupational risk factors, for example, asbestos, must be considered to define high-risk lung cancer populations.^16^ The lung cancer detection rate in a lung cancer screening program for workers at high risk for lung cancer because of a combination of occupational exposures and smoking was similar to that observed in the NLST.^17^ In 2022, Markowitz reported the importance of promoting lung cancer screening with low-dose chest CT for workers exposed to asbestos.^18^ In France, three academic societies (occupational health, pneumology, and radiology) recommended implementing such an organized lung cancer screening program for subjects exposed or occupationally exposed to pulmonary carcinogens at high risk of lung cancer.^19^ In response to that recommendation, the LUng Cancer Screening Occupational exposure (LUCSO) study^20^ was designed as a feasibility study for organized lung cancer screening for subjects with occupational exposure to pulmonary carcinogens in eight administrative divisions in France.

Herein, we report the program’s entry findings.

## Methods

The LUCSO study is a prospective, multicenter cohort study aimed at implementing a lung cancer screening program in populations exposed to lung carcinogens across eight departments in France. This manuscript reports on the feasibility study conducted in the first two departments—Gironde and Val-de-Marne.

### Population

Subjects aged 55 to 74 years, with sufficient exposure to occupational carcinogens in IARC group 1 and smokers or ex-smokers for less than 15 years, were eligible for inclusion (Table 1). We selected carcinogens classified by the IARC as definite lung carcinogens with sufficient evidence for lung cancer, and that could be found in the workplace, as defined in the French recommendation.^21^ The noninclusion criteria were clinical signs of lung cancer, a prior lung cancer, serious co-morbidities threatening short-term life expectancy, no exposure to occupational lung carcinogens according to predefined criteria, ongoing inclusion in another prospective cohort study, chest CT scan within the last year, nonexposure or insufficient exposure to tobacco, or ex-smoker for more than 15 years.

**Table 1:** Inclusion Criteria

Active or <15 y Former Smoker	Occupational Exposure	Cumulative Exposure Level^a^
≥30 PY	Asbestos	Intermediate
≥30 PY	Asbestos	High with duration <5 y
≥20 PY	Asbestos	High with duration ≥5 y
≥30 PY	Other carcinogens^b^	
≥20 PY	2 other carcinogens^b^	
≥10 PY	≥3 other carcinogens	

PY, pack-years.

a From the definition used by the 1999 French consensus conference experts on the follow-up in workers exposed to asbestos. High exposure: confirmed, high and continued exposure for ^3^1 year; examples: working in the manufacture or transformation of materials including asbestos and their equivalents during manipulation/handling of materials or equipment likely to discharge asbestos fibers (e.g., fireproofing, naval construction); confirmed, high, and intermittent exposure for ≥10 years (e.g., Manipulation or handling operators on heavy goods vehicle brake systems, cutting of asbestos cement); intermediate exposure: all other documented significant occupational exposure situations. Most of these situations involve work with materials or equipment likely to discharge asbestos fibers.

b Aluminum production, coal gasification, coal tar pitch, coke production, x-rays and gamma rays, radon, iron-ore mines, plutonium, steel foundries, painter’s materiel, rubber production, chromium (VI) compounds, beryllium, cadmium and its compounds, bis(chloromethyl) ether, chloromethyl methyl ether, metal cobalt with tungsten carbide, and welding fumes. Special cases: asbestos (if asbestosis is present, 20 PY of smoking are necessary; if pleural plaques are present, 30 PY of smoking are necessary); crystalline silica (silicosis is necessary to integrate the high-risk group for lung cancer, independently of exposure duration); diesel engine exhaust fumes (a high level of exposure defined by employment in underground mines, tunnel construction, or underground mine maintenance is necessary to integrate the high-risk group for lung cancer).

### Identification of Subjects With Current or Past Occupational Exposure to Lung Carcinogens

The study initially targeted subjects aged 65 to 74 years in two French regional counties to test the program’s feasibility. All subjects in that age group received an invitation letter from the Regional Cancer Screening Coordination Center, in monthly waves over 1 year. That letter included information about the screening program, eligibility criteria, a simplified two-page self-reporting questionnaire about workplace exposures and smoking history, and a prestamped envelope. An industrial hygienist and an occupational physician analyzed returned questionnaires and classified respondents as eligible, not eligible, or eligibility uncertain. For the latter case, the industrial hygienist called the subject to obtain more details of the individual’s work history. For patients not eligible, according to smoking history and risk-factor exposure level, a letter was sent giving them advice on quitting tobacco, the possibility of inclusion in a general population lung cancer screening program, and/or the possibility of postprofessional follow-up in an occupational health center. Eligible subjects were invited to a consultation in an occupational health clinic (one center in each county). During this consultation, eligibility criteria were checked, including smoking status, cumulative level of exposure to various occupational risk factors, and other risk factors. Subjects were offered inclusion in the LUCSO program, based on a low-dose CT scan at baseline and spirometry flow-volume loop, followed by an annual CT scan at 1 and 2 years and telephone follow-up at 3 years. They received information about the program, the risks of detecting abnormalities on the CT scan, diagnosing tumors that may never have caused clinical symptoms (overdiagnosis) or affected survival (rare indolent form) and/or radiation exposure from repeated chest CT scans, and the benefits of stopping smoking. The French Institute for Health Education and Prevention file “Smoking Cessation Consultations” was completed during the initial examination, and subjects were referred to stop-smoking consultations, during which they were assessed and managed according to national guidelines.^22^ Each participant had a low-dose, multislice volumetric CT scan without contrast injection in deep inspiratory apnea, with the arms positioned over the head from the apex to the pleural cul-de-sacs, according to American College of Radiology quality-control requirements.^23^ A specifically trained senior thoracic CT radiologist^24^ read the scan according to the Fleischner Society recommendations for the measurement of incidental nodules and national recommendations.^25^ Positive and/or incidental pulmonary or extrapulmonary lesion (chronic obstructive pulmonary disease, diffuse interstitial lung disease, etc.) detection could lead to referral for management by a pulmonologist.^8^^,^^26^, ^27^, ^28^

### Statistical Analyses

The numbers of individuals invited, self-questionnaires returned, eligible individuals, participants included, and individuals eligible for postprofessional follow-up observation aspects were analyzed. The following information was collected for included subjects: sociodemographic, medical, and lifestyle characteristics including age, sex, medical or surgical history, chronic cough, exertional dyspnea, smoking status, and PY; occupational exposures throughout their careers; average number of jobs held (duration^3^ 6 months); description of occupations and industries (according to the International Standard Classification of Occupations codes) in which more than 10 participants worked in the same sector or profession; percentages of workers exposed to each confirmed carcinogen, to two or three or more carcinogens; and screening low-dose chest CT scan findings including rates of negative, uncertain, and positive images, with the percentages of positive and negative follow-up results at 3 months for uncertain scans; and the lung cancer diagnosis rate across all participants screened with positive scan results.

### Ethical and Legal Aspects

Upon enrolling in the study, the investigator explained the study information sheet to the participants and provided them with a copy. The participants also signed a consent form. The clinical study received favorable approval from the Committee for the Protection of Persons Sud-Est-I Ethics Committee on February 5, 2017 (number 2017-A02275-48). The data system used for this research complies with French regulations (amended Data Protection Act) and European regulations (General Data Protection Regulation) and received approval from the French Data-Protection Agency (CNIL Decision DR-2019-015) in August 2018.

## Results

**Figure 1:**
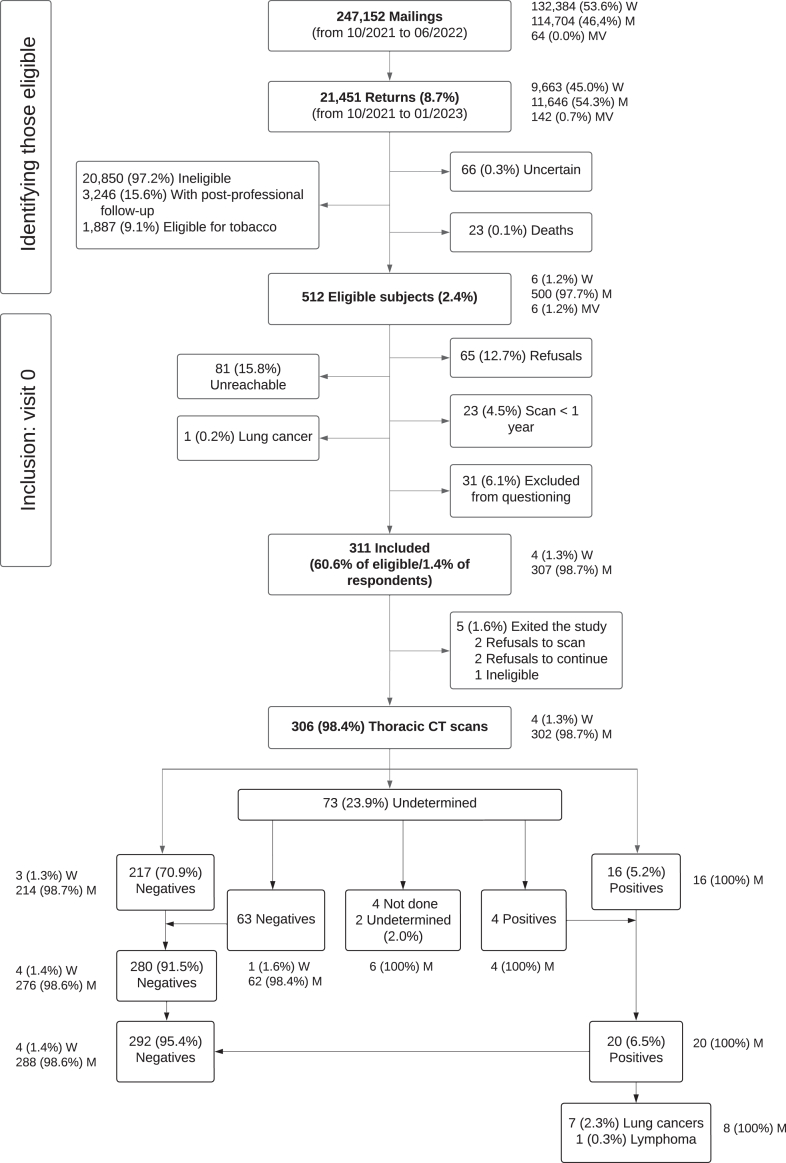
Flowchart of subject invitations and inclusion in the feasibility phase of the LUng Cancer Screening Occupational exposure study. CT, computed tomography; M, men; MV, missing values; W, women.

Between February 2022 and June 2023, 247,152 letters were sent to all the following individuals aged 65 to 74 years: 21,451 self-questionnaires (8.7%) were returned and 513 subjects (2.4%) were deemed eligible (Fig. 1). Among the 311 included (1.4% of respondents; 60.6% of eligible), 307 (98.7%) were men, and five were excluded for refusals and/or ineligibility, leaving 306 participants for analysis. After occupational physicians assessed occupational exposure during the inclusion in-person consultation, 90.8% were exposed to asbestos, among 69.9% for more than 10 years (Table 2). The three other primary nuisances found were crystalline silica (70%), welding fumes (48%), and painter’s materiel (35%). Respectively, 28.1% and 68.3% had been exposed to more than two confirmed lung carcinogens during their careers, with consumption of tobacco for at least 20 PY and three confirmed lung carcinogens during their careers, with consumption of tobacco for at least 10 PY.

**Table 2:** Known Occupational Exposures to Lung Carcinogens for 306 Participants

All DurationLung Carcinogen	All Duration	Duration Exposure >10 y
	n (%)	n (%)
Asbestos	278 (90.8)^a^	214 (69.9)
Silica	214 (69.9)	-
Welding fumes	147 (48.0)	105 (34.3)
Painter's materiel	107 (35.0)	78 (25.5)
Diesel exhaust fumes	84 (27.5)	62 (20.3)
Chromium VI compounds	31 (10.1)	26 (8.5)
Nickel compounds	31 (10.1)	27 (8.8)
Soot	41 (13.4)	31 (10.1)
Other PAHs	7 (2.3)	5 (1.6)
Cobalt metal with tungsten carbide	18 (5.9)	15 (4.9)
Cadmium and its compounds	12 (3.9)	6 (2.0)
Coal pitch	9 (2.9)	4 (1.3)
X-rays or gamma-rays	5 (1.6)	4 (1.3)
Beryllium	7 (2.3)	6 (2.0)
Arsenic and derivatives	7 (2.3)	4 (1.3)
Iron mines	1 (0.3)	–/0
Radon	4 (1.3)	1 (0.3)
Bis(chloromethyl) ether, chloromethyl methyl ether	2 (0.7)	2 (0.7)
Cast iron and steel foundry	5 (1.6)	3 (1.0)
Aluminum production	–/0	–/0
Coal gasification	–/0	–/0
Rubber manufacturing	–/0	–/0
Coke production	–/0	–/0
Plutonium	–/0	–/0

PAH, polycyclic aromatic hydrocarbons

a Sixteen were exposed <10 years with high cumulative exposure level.

Of the 311 subjects included, 306 (98.4%) had a baseline CT scan; 302 (98.7%) were men; median age 69.0 years; 45% were active smokers at inclusion, with average tobacco consumption of 38.9 (10–112) PY. Among those who no longer smoked at inclusion, the median duration since quitting smoking was 8.4 years. More than 96% of the participants had a medical or surgical history (Table 3).

**Table 3:** Medical History of 306 Participants

Medical History	n	%
High blood pressure	118	38.6
Diabetes	44	14.4
Hypercholesterolemia	42	13.7
Chronic obstructive broncho pneumopathy	17	5.6
Asthma	14	4.6
Sleep apnea	14	4.6
Stroke	11	3.6
Pneumonia	7	2.3
Transient ischemic attack	5	1.6
Chronic bronchitis	5	1.6
Pneumothorax	5	1.6
COVID-19	4	1.3
Pulmonary embolism	1	0.3

COVID-19, coronavirus disease 2019.

Participants held a mean (± SD) of 5.2 jobs (± 2.2), range; 1 to 16. The most frequently occupied sectors were 49% in specialized construction work; 48% held at least one public administration and defense job; 40% worked in education (Supplementary Table 1). Among the most held professions (Supplementary Table 2), nearly 46% of participants worked at least once in skilled building trades and related professions (excluding electricians), 38% in skilled metallurgy, mechanical construction trades, and/or related professions; 27% had professions related to the armed forces.

Of the 139 smokers, 52 (37.4%) agreed to schedule a stop-smoking consultation, and 45 (86.5%) attended. Among the 306 participants with entry screening scans, respective negative, positive, and indeterminate finding rates were 217 (70.9%), 16 (5.2%), and 73 (23.9%). For those with indeterminate results, four follow-up CT scan findings (5.5%) at 3 months were deemed positive (Fig. 1). Among the 20 subjects whose scan findings were classified as positive, seven (2.3%) were diagnosed with lung cancer (three stage IA, three stage IIA, and one stage IIB in one participant). The false-positive rate was 4.2%.

The other anomalies identified on the screening scan are detailed in Table 4. One lymphoma was also diagnosed. In spirometry, 20% had an obstructive ventilatory disorder (forced expiratory volume in 1 second/forced vital capacity <0.7).

**Table 4:** Abnormalities Detected on 306 Screening Low-Dose Chest Computed Tomography Scans (a Subject Can Have > One Abnormality)

Abnormality Observed	n (%)
Severe coronary artery calcifications	85 (27.8)
Thoracic aorta aneurysm	20 (6.5)
Emphysema	139 (45.4)
Respiratory disease	94 (30.7)
Interstitial lung disease	24 (7.8)
Pleural plaques	9 (2.9)

## Discussion

The entry-level findings of this ongoing feasibility study in France provided new real-life information on the implementation of lung-cancer screening in a population with occupational exposure to lung carcinogens. After an exclusively age-based invitation, 8.7% of the subjects returned their self-reporting questionnaires; 2.4% were eligible, and 60.6% of those eligible (1.4% of respondents) were included. Rates of positive screening finding images, lung cancer diagnoses, and false positives were, respectively, 6.5%, 2.3%, and 4.2%. Selection based on occupational exposure led to 98.7% of those eligible being men.

Among these participants, predominant asbestos exposure (91%) was associated with co-exposures (70% to crystalline silica and 48% to welding fumes); 45% were active smokers at inclusion, and 37% agreed to schedule a consultation; 73% among those who attended the consultation expressed a desire to quit smoking. CT-scan screening visualizing severe coronary artery calcifications and emphysema were identified on images in 27% and 47% of the subjects, respectively. According to the French SUMER (Surveillance médicale des expositions des salariés aux risques professionnels) 2017 survey conducted in randomly selected, professionally active subjects with estimation of the current exposure in the week preceding the interview by the occupational physician, the most prevalent lung carcinogen exposure was to diesel exhaust fumes, followed by crystalline silica and asbestos.^29^ However, asbestos exposure was apparently underestimated in the SUMER study because of discontinuous exposure for the course of a working life, and because occupational physicians may also be unaware of exposure settings.

Among the subjects who returned the questionnaire, 2.4% were eligible. That response rate may seem low, but it is indeed higher than expected on the basis of ICARE study data (case–control lung cancer study in a general population in France^30^); for the same age group and tobacco- and asbestos-exposure levels, the expected eligibility rate was 1%. Our eligible-subject rate was higher, perhaps because those not eligible for screening were less likely to respond, and the ICARE study only considered individuals exposed to tobacco and asbestos. Literature findings indicate that participation in lung cancer screening is linked to participants’ perception of their lung cancer risk, which might be the reason smokers and those exposed to lung carcinogens in the workplace were more likely to respond than the general population.^31^ However, the population-wide screening approach maintains a low eligibility rate. At present, no database has integrated tobacco use and occupational exposure data to identify the LUCSO target population in France.

The lung cancer diagnosis rate during this initial phase of inclusion was 2.3%, which is higher than those later reported for the NLST (1%) and NELSON (0.9%) study,^4^^,^^5^ thereby suggesting the targeted occupational exposure and smoking population has, as expected, a higher lung cancer risk than the smokers in those previous randomized trials. Furthermore, it is known that the selected population exposed to occupational lung carcinogens and cigarette smoke is at greater risk of lung cancer than are those exposed to each factor individually.^32^ However, the definition of high-risk populations established by several risk-prediction models for lung cancer developed since the NLST results became available, but only three included asbestos exposure as a risk factor^33^, ^34^, ^35^ and address it only superficially through binary questions (yes/no) that fail to accurately assess the duration and intensity of lung carcinogen exposure. Few studies on lung cancer screening are ongoing in populations with occupational carcinogen exposure, and they only address workers exposed to asbestos and US workers on nuclear weapons. A review of the literature concluded that screening asbestos workers with no other risk factors for lung cancer, such as tobacco smoking, increased the low detection rate.^36^ On the basis of a population exposed to asbestos screened with annual low-dose CT scans, Brims et al. found that 65% of the subjects diagnosed with lung cancer would not have been eligible for screening based solely on smoker status.^37^ The lung cancer screening findings of US workers on nuclear weapons were similar to those reported for the NLST.^17^ As recommended by the Collegium Ramazzini,^32^ research to better define the target population of workers at high risk of lung cancer should be continued. Moreover, although our initial screening rate is higher than those reported for the NLST and NELSON trial, the seven lung cancers detected were in early stages, indicating the effectiveness of this screening approach.

In our population, 45% of participants remained smokers. It is important that lung cancer screening for smokers also serves as an opportunity to encourage quitting smoking.^38^ At this time, we do not have data on stopping rates, but 37% of smokers agreed to a consultation with an addictology specialist, and among those, 73% wanted to quit. Moldovanu et al. described reported smoking-cessation rates ranging between 7% and 23%, with 55% and 85% of participants reporting that screening played an important role in their decision to quit smoking.^39^

Our study results also confirmed the high rate of cardiovascular disease, including severe coronary artery calcifications, which can be detected as part of lung-cancer screening. The older age and predominance of men in the LUCSO study may explain the particularly high rate of severe coronary artery calcium in this study. This rate is higher than that of several studies performed in the general population using criteria based solely on smoking, such as NLST, NELSON, or, in France, LUMASCAN (20%).^40^ Our screening results also open the way to obtaining recognition of carcinogen exposure as an occupational disease in France. Inclusion in this initial analysis of LUCSO entry findings was also intended to facilitate medical-social processes, particularly the recognition of work-related diseases for all participants with conditions linked to major occupational exposure, including not only the identified lung cancers but also benign conditions resulting from occupational exposures, such as pleural plaques, the most common condition after asbestos exposure. It remains too early to know whether participants in LUCSO will benefit from occupational disease recognition. Future results could enable evaluation of this possibility.

For this study, we chose to invite the entire general population on the basis of age alone to this organized screening, given no database in France enables us to identify smokers or ex-smokers with occupational exposure to lung carcinogens. From an ethical point of view, this screening could therefore be offered to the entire target populations of different ages. Nonetheless, this recruitment method probably highly selected our population, which does not reflect the general population’s exposure to lung carcinogens in the workplace. It is indeed possible that the people who felt most concerned by the study responded more than others, which could have selected subjects at higher risk of lung cancer and thus led to a high rate of lung cancer in this study.

## Conclusion

Collectively, this feasibility study provided new insights before considering nationwide implementation. Such entry screening addresses a significant social concern, with guidelines published in 2016 in France, including recommendations for screening of other populations considered at high risk of lung cancer because of co-exposure to smoking and occupational carcinogens. Entry-level findings confirmed the high risk of lung cancer in the selected population, and screening efficacy in detecting early stages and visualizing several other unknown abnormalities on CT scan, enabling subsequent initiation of active prevention, especially for cardiovascular disease.

## CRediT Authorship Contribution Statement

**Fleur Delva:** Conceptualization, Data curation, Formal analysis, Funding acquisition, Investigation, Methods, Project administration, Resources, Software, Supervision, Validation, Visualization, Writing - original draft, Writing - review and editing.

**Sébastien Gendarme:** Data curation, Investigation, Validation, Visualization, Writing - review and editing.

**Luca Boudet:** Data curation, Investigation, Validation, Visualization, Writing - review and editing.

**Maeva Zysman:** Data curation, Investigation, Validation, Visualization, Writing - review and editing.

**Claire Fuhrman:** Data curation, Investigation, Validation, Visualization, Writing - review and editing.

**Pierre-Yves Brillet:** Data curation, Investigation, Validation, Visualization, Writing - review and editing.

**Patrick Brochard:** Conceptualization, Funding acquisition, Methods, Supervision, Validation, Visualization, Writing - review and editing.

**Christophe Paris:** Conceptualization, Funding acquisition, Methods, Resources, Supervision, Validation, Visualization, Writing - review and editing.

**Olivier Bylicki:** Conceptualization, Funding acquisition, Methods, Resources, Supervision, Validation, Visualization, Writing - review and editing.

**Véronique Le Denmat:** Conceptualization, Funding acquisition, Methods, Resources, Supervision, Validation, Visualization, Writing - review and editing.

**Brice Loddé:** Validation, Visualization, Writing - review and editing.

**Milia Belacel:** Project administration, Validation, Visualization, Writing - review and editing.

**Nadia Abdessemed:** Data curation, Investigation, Validation, Visualization, Writing - review and editing.

**Murielle Despagne:** Data curation, Investigation, Validation, Visualization, Writing - review and editing.

**Kellian Jaunas:** Data curation, Formal analysis, Funding acquisition, Software, Validation, Visualization, Writing - review and editing.

**Aude Lacourt:** Conceptualization, Data curation, Funding acquisition, Investigation, Methods, Resources, Supervision, Validation, Visualization, Writing - review and editing.

**Bénédicte Clin:** Conceptualization, Funding acquisition, Methods, Resources, Supervision, Validation, Visualization, Writing - review and editing.

**Jean-François Gehanno:** Conceptualization, Funding acquisition, Methods, Resources, Supervision, Validation, Visualization, Writing - review and editing.

**François Laurent:** Conceptualization, Funding acquisition, Methods, Resources, Supervision, Validation, Visualization, Writing - review and editing.

**Gael Dournes:** Validation, Visualization, Writing - review and editing.

**Simone Mathoulin-Pélissier:** Conceptualization, Funding acquisition, Methods, Resources, Supervision, Validation, Visualization, Writing - review and editing.

**Christos Chouaïd:** Conceptualization, Funding acquisition, Methods, Resources, Supervision, Validation, Visualization, Writing - original draft, Writing - review and editing.

**Jean-Claude Pairon:** Conceptualization, Formal analysis, Funding acquisition, Methods, Project administration, Resources, Supervision, Validation, Visualization, Writing - original draft, Writing - review and editing.

## Data Availability Statement

Data are unavailable to access; they contain confidential information.

## Disclosure

Chouaïd received consulting fees and support for attending meetings or travel from AZ, BI, GSK, Roche, Sanofi Aventis, BMS, MSD, Lilly, Novartis, Pfizer, Takeda, Bayer, Pierre Fabre, Daichi, and Amgen. Dr. Gendarme and Mrs. Belacel receive support from the French “Association de Recherche sur le Cancer” (ARC) (by the institution). Mrs. Belacel received support from the French “Association La Ligue contre le cancer” (by the institution). Bylicki received payment or honoraria for lectures, presentations, speakers’ bureaus, manuscript writing, or educational events from MSD, Astra Zeneca, and Janssens and Janssens and support for attending meetings or travel from MSD, Astra Zeneca, and Pfizer. Loddé received payment or honoraria for lectures, presentations, speakers’ bureaus, manuscript writing, or educational events from Novo Nordisk, Sanofi, and Léo Pharma pharmaceutics. The remaining authors declare no conflict of interest.

## Footnotes

Note: To access the supplementary material accompanying this article, visit the online version of the *JTO Clinical and Research Reports* at www.jtocrr.org and at https://doi.org/10.1016/j.jtocrr.2026.100995.
