# Supplementary material for: Organized Lung Cancer Screening of Subjects Occupationally Exposed to Lung Carcinogens: The LUng Cancer Screening Occupational exposure Study Entry Findings
Source: JTO Clin Res Rep. 2026 Mar 28;7(6):100995. doi: 10.1016/j.jtocrr.2026.100995 (PMC13206741; doi:10.1016/j.jtocrr.2026.100995)
Supplement: Table S1 [file mmc1.docx]

**Table S1**

Description of the Activity Sectors Involved during participant’s professional careers (if ≥10 subjects).

| **Activity sector** | **n** | **%** |
| --- | --- | --- |
| Specialized construction work | 150 | 49.0 |
| Public administration and defense; compulsory healthcare coverage | 146 | 47.7 |
| Student | 122 | 39.9 |
| Trade and repair of motor vehicles and motorcycles | 61 | 19.9 |
| Building construction | 60 | 19.6 |
| Retail trade, except motor vehicles and motorcycles | 31 | 10.1 |
| Land transport and pipeline transport | 29 | 9.5 |
| Metal product manufacturing, except machinery and equipment | 24 | 7.8 |
| Machinery and equipment repair and installation | 21 | 6.9 |
| Crop and animal production and related services | 20 | 6.5 |
| Manufacture of other transport equipment | 18 | 5.9 |
| Employment-related activities | 16 | 5.2 |
| Activities for human health | 16 | 5.2 |
| Automobile industry | 16 | 5.2 |
| Wholesale trade, except motor vehicles and motorcycles | 14 | 4.6 |
| Other non-metallic mineral-product manufacturing | 13 | 4.2 |
| Food Industries | 12 | 3.9 |
| Civil engineering | 11 | 3.6 |
| Metallurgy | 11 | 3.6 |
| Manufacture of machinery and equipment not elsewhere | 11 | 3.6 |
| Restauration | 10 | 3.3 |

**Table S2**

Description of the Participants’ Occupations During Their Professional Careers (if ≥10 subjects).

| **Occupations** | **n** | **%** |
| --- | --- | --- |
| Skilled building trades and related occupations, except electricians | 142 | 46.4 |
| Skilled trades in metallurgy, mechanical engineering and related occupations | 117 | 38.2 |
| Other members of the armed forces | 83 | 27.1 |
| Intermediate science and technology professions | 81 | 26.4 |
| Electrical and electrical engineering professions | 50 | 16.3 |
| Laborers in mining, construction and public works, manufacturing and transport industries | 41 | 13.4 |
| Drivers of heavy lifting and shunting vehicles and equipment | 40 | 13.1 |
| Technical science specialists | 24 | 7.8 |
| Operators of machines and fixed installations | 21 | 6.9 |
| Food, woodworking, clothing and other skilled industrial and craft occupations | 21 | 6.9 |
| Farmers and skilled commercial agricultural workers | 17 | 5.6 |
| Directors and executives, production and specialized services | 17 | 5.6 |
| Intermediate professions, finance and administration | 17 | 5.6 |
| Garbage collectors and other unskilled workers | 16 | 5.2 |
| Assembly workers | 16 | 5.2 |
| Chief executive officers, senior managers and members of the executive and legislative bodies | 14 | 4.6 |
| Traders and sellers | 11 | 3.6 |
| Administrative and commercial managers | 11 | 3.6 |
| Housekeepers | 10 | 3.3 |
| Protective and security services personnel | 10 | 3 .3 |
| Direct personal services occupations | 10 | 3.3 |
| Healthcare-associated professions | 10 | 3.3 |
| Associate professionals in legal, social and related services | 10 | 3.3 |
